# Cardiotrophin-1: a new player in energy metabolism with potential therapeutic application

**DOI:** 10.18632/aging.100359

**Published:** 2011-08-14

**Authors:** Matilde Bustos, María J Moreno-Aliaga, Jesús Prieto

**Affiliations:** ^1^ Division of Hepatology and Gene Therapy, Center for Applied Medical Research (CIMA). University of Navarra, 31008 Pamplona, Spain; ^2^ Department of Nutrition, Food Sciences, Physiology and Toxicology. University of Navarra, 31008 Pamplona, Spain

The incidence of obesity and associated co-morbidities such as insulin resistance, dyslipidemia and cardiovascular diseases has reached epidemic proportions worldwide. As a result, new therapeutic options for the treatment of these conditions are clearly warranted. With the failure of leptin as anti-obesity therapy, recent research has focused on the gp130 receptor ligands, in particular CNTF, as potential therapeutic agents for obesity [1]. This cytokine induces weight loss and improves glucose tolerance [2] but its body weight lowering activity in clinical trials was limited [3]. On the other hand, the effects of IL-6 (another gp130 ligand) on energy metabolism remain controversial. Although IL-6 has been proposed to increase insulin resistance, the discovery that IL-6 is a myokine produced and released from skeletal muscle during exercise, led many to challenge this concept as insulin action is enhanced immediately after exercise [4]. In any case clinical use of IL-6 is hampered by its potent pro-inflammatory properties.

A recent publication by our group showed that cardiotrophin-1 (CT-1), another member of the gp130 family of cytokines, plays a key role in energy metabolism [5]. This cytokine which is constitutively expressed in muscle, heart, liver and white adipose tissue (WAT) has been previously shown to exert important cytoprotective activities. In particular we have reported that CT-1 is a natural defence of the liver against apoptosis being capable of protecting the liver against immunological and ischemic insults [6,7]. In analyzing the metabolic effects of CT-1 we observed that CT-1 is a nutritional-regulated gene that is upregulated by fasting and downregulated by refeeding. Also we found that *ct-1* null mice exhibited a marked reduction of energy expenditure and that these animals developed mature-onset obesity, insulin resistance and hypercholesterolemia, mimicking the human metabolic syndrome. Interestingly, we observed that administration of recombinant CT-1 (rCT-1) decreased body weight by reducing food intake and enhancing energy expenditure. Moreover, this treatment induced a marked remodeling of WAT as result of increased lipolysis, enhanced fatty acid oxidation and stimulation of mitochondrial biogenesis. These changes, together with upregulation of genes typifying brown fat phenotype resulted in a sharp decrease of adipocyte size and increase oxygen consumption by these cells. On the other hand, CT-1 therapy decreased blood glucose in an insulin independent manner and increased insulin-stimulated AKT phosphorylation in muscle. CT-1 also promoted fatty acid oxidation in muscle, an effect which was not observed in *AMPKα2^−/−^* mice. In agreement with all these metabolic effects, CT-1 treatment was able to correct obesity and associated diabetes in animal models of genetic and acquired obesity. Thus, CT-1 has emerged as a new player in the control of energy metabolism with potential application in the treatment of obesity and type 2 diabetes.
